# Barriers to Diagnosis of Postpartum Depression among Younger Black Mothers

**DOI:** 10.21203/rs.3.rs-2500330/v1

**Published:** 2023-02-16

**Authors:** Megana Dwarakanath, Fahmida Hossain, Phoebe Balascio, Mikaela C. Moore, Ashley V. Hill, Natacha M. De Genna

**Affiliations:** University of Pittsburgh Medical Center; University of Pittsburgh Medical Center; University of Pittsburgh Medical Center; University of Pittsburgh Medical Center; University of Pittsburgh Medical Center; University of Pittsburgh Medical Center

**Keywords:** postpartum depression (PPD), adolescent, mothers, stigma, equity, Black women

## Abstract

**Objective:**

The objective of this study was to qualitatively examine coping mechanisms and desired supports in pregnant and birthing Black and biracial adolescent and young adult women during the COVID-19 pandemic.

**Method:**

Black and biracial participants ages 16–23 were recruited for virtual individual semi-structured interviews. Participants (n=25) were asked about pre- and post-natal experiences with the healthcare system, effects of the pandemic, and participants’ experiences of or desires for ideal care within the healthcare system. Interviews were transcribed verbatim and coded for qualitative analysis using nVivo. Discussions around postpartum mental health evolved organically when asked about how participants were coping postpartum.

**Results:**

Nearly half the interviewees reported mental health symptoms consistent with postpartum depression (PPD). Of the 11 interviewees who reported mental health symptoms consistent with PPD, 2 were afraid to disclose their symptoms to a healthcare provider due to fear of child protective services involvement and their belief they would be treated unfairly because of their race.

**Conclusion:**

Clinicians who care for Black and biracial adolescent and young adult mothers must be particularly attuned to structural barriers for appropriate screening and treatment of postpartum depression. Expanding investigations of intersectional influences on young mothers’ perinatal health and PPD are needed.

## 1. Introduction

While postpartum depression (PPD) is known to affect adolescents at double the rates of adults [1], literature in this field is limited among adolescent and young adult pregnancies. Adolescence is a period of significant social, emotional, and physical transition during which the addition of motherhood can create further stressors, in part due to age related stigma [2, 3]. In fact, individuals who transition to motherhood at younger ages report more depressive symptoms [4]. Young mothers must also navigate the new role as a parent within an existing structure of peer relations with peers that may not have similar experiences and responsibilities [5]. Adolescent mothers may be at increased risk of facing socioeconomic challenges including lower income, single parent status [6] as well as social and emotional risk factors such as intimate partner violence, family conflict, low self-esteem and fewer social supports [7, 8]. These risk factors may be particularly compounded by structural inequities and racism, as literature has demonstrated that Black and Hispanic women in the general population may be more likely to suffer from postpartum depression [9]. They are also less likely to initiate postpartum mental health care as well as follow-up treatment for postpartum depression [10].

During the COVID-19 pandemic, rates of postpartum depression escalated dramatically. According to one meta-analysis, the prevalence of postpartum depression was 34% during COVID-19, compared to 10% in developed countries and 21–26% in developing countries prior to the pandemic [11]. Several risk factors associated with postpartum depression likely became exacerbated with the pandemic, including fewer social supports due to isolation and lower income [12], contributing to the marked increase in depressive symptoms, especially for minority communities who were disproportionately affected by the pandemic [13]. Pregnant Black women have reported more stress, financial strain, and concerns about their medical care during the pandemic compared to pregnant non-Hispanic white women [14, 15].

With the multiplicity of risk factors that predispose minority mothers to postpartum depression, understanding and intervening to treat mental health sequelae of adolescent pregnancy is paramount for both maternal and infant outcomes. Unaddressed PPD could have potentially devastating consequences for both infants and mothers including poor neurodevelopmental outcomes in infants, and low self-esteem, intimate-partner violence, suicidal ideation, difficulties with parenting skills, substance use disorder, and persistent mental illness after the postpartum period in mothers [14]. A multiplicity of factors may indeed hinder accurate self-reporting, especially among Black others.

The results of one study with low-income Black mothers in New York City suggest that this population may be underdiagnosed and undertreated for postpartum depression. In this pilot intervention to prevent depression among the general population (i.e., not specific to adolescents and young adults), clinical social work staff noted that patients were reluctant to acknowledge the impact of stress and mental health on screening tools, due to stigma as well as not having practice or not knowing the language to express mental health concerns. One of the social workers in the study stated that patients were concerned that disclosure about mental health issues would lead to child protective service involvement. Another social worker noted that participants would screen negative on a PHQ-9, only to disclose symptoms of stress and concern after building trust with a provider. Only 15% of participants scored 10 or above on the PHQ-9 during the time period of the intervention, a low percentage given the financial stressors and race based discrimination that impacted the sample. Interestingly, in the same intervention, many patients deferred services for postpartum depression, citing that they could handle the stress or had support from their church. Only two of the 30 eligible participants for intervention followed up with the program [16].

As underreporting of PPD may be high among low-income Black mothers, it is paramount to better understand the experiences of Black adolescent and young adult pregnancies, particularly those demonstrating symptoms of PPD, and to determine barriers to treatment. This is critical to ameliorating gaps in care for the most vulnerable patients during a challenging life transition. Therefore, the objective of this study was to qualitatively examine the pregnancy experiences of adolescent and young adult Black and biracial women during the novel coronavirus pandemic in one geographic region, to assess coping mechanisms and desired supports.

## 2. Methods

### 2.1. Setting and Context

This qualitative study was conducted as a supplement to an existing project, the YoungMoms study (R01DA046401), a longitudinal cohort study designed to examine perinatal cannabis and tobacco co-use in young people and associated infant outcomes. The purpose of the supplemental study was to qualitatively assess the impact of COVID-19 on the experiences of young Black and biracial mothers, particularly in regard to substance use. Pregnant persons ages 13–21 were recruited at or before a prenatal visit. Participants who completed the baseline survey and were < 14 weeks gestation were recruited for the longitudinal study. YoungMoms participants who identified as Black or Biracial in the survey but who were not enrolled in the longitudinal study were recruited (by telephone, email or text message) to participate in 45–60-minute semi-structured, in-depth interviews for this qualitative study. IRB approval was obtained after review by the University of Pittsburgh Office of Research Protections. We followed Consolidated Criteria for Reporting Qualitative Research (COREQ) guidelines [17].

### 2.2. Interview Guide Development

The interview guide was developed by the PI of the YoungMoms study in collaboration with a Co-Investigator with expertise on Black women’s reproductive health and further refined with feedback from the interviewers (ND & AH). Open-ended questions focused on the impacts of structural and racial discrimination as well as the COVID-19 pandemic on the pregnancy and postpartum experiences of Black and Biracial adolescents and young adults (see Supplement). Participants were asked about pre- and post-natal experiences with the healthcare system, effects of the pandemic (including social isolation, vaccination status, and infection; coping mechanisms and substance use); obstetric racism; and participant-driven notions and experiences of ideal care within the healthcare system. Interviews lasted roughly 45–60 minutes. Although not the intended purpose of this investigation at inception, many of the participants (n = 11) described some form of postpartum depression, creating the impetus for this study and the desire to expand the research beyond the initially described purpose.When interviewing the participants about their postpartum physical and mental health experiences to understand postpartum experiences more broadly among Black and biracial young mothers, participants organically disclosed symptoms consistent with postpartum depression.

### 2.3. Data Collection

A research team member recruited eligible individuals by telephone, provided details about the study, and answered any questions from those interested in participating. Participants provided verbal informed consent and were offered $50 on a gift card for their participation. Interviews were conducted remotely over Zoom by two interviewers (F.H., M.D.). A clinician and a researcher with experience conducting qualitative research conducted the interviews (F.H. and M.D.), which allowed for a balance of interviewing strategies and approaches in the semi-structured interview format. The interviews were recorded and transcribed with the Zoom transcription feature. Interviews were then de-identified and the transcripts were manually edited by a student and two of the authors (MM, FH) to reflect the audio recording. Interviews were conducted until the research team concluded thematic saturation was achieved. Demographic data were collected from the survey for the parent study.

### 2.4. Data analysis

The research team used thematic content analysis to identify major themes and subthemes, with the preliminary codebook developed from 5 interviews. Two teams of investigators (F.H. and M.D; P.B. and M.M) coded the interviews separately using qualitative analysis software (NVivo) Code books were then compared for agreement and finalized through consensus, with iterative refinement of the codebook as more interviews were completed and coded.

## 3. Results

We interviewed 25 study participants (88% Black, 12% Biracial) who ranged from 16–23 years of age at the time they participated in this study (March-July 2022). This was the first pregnancy for most participants (n = 15). Additional sociodemographic characteristics are presented in Table 1. Out of the eleven participants reporting symptoms consistent with postpartum depression, only two participants reported telling their health care provider about these symptoms, and only one reported that they were subsequently connected to resources. Three participants specifically reported fear of child protective services as a reason for not reporting.

Four major themes emerged from the narratives of participants: (1) Symptomatology Consistent with Postpartum Depression (2) Self-Blame Around Postpartum Mental Health Sequelae; (3) Fear Admitting Postpartum Mental Health Sequelae to a Health Care Provider, and (4) Social Support and Affirmation as Critical Elements in Coping with Postpartum Mental Health Sequelae.

### 3.1. Theme 1: Symptomatology Consistent With Postpartum Depression

Despite the interview guide not explicitly asking about postpartum depression, of 25 participants, 11 described symptomatology consistent with postpartum depression while describing their postpartum experiences. One interviewee described wanting to “run away from it all:”

But when I say that I don’t know what I mean… I don’t know if I mean like just literally physically, stop doing everything like just stay in one spot, I don’t know if that means that I wanna maybe pack my own bags up and run away from all of my problems or your problems or life. My life. This life. You know I know genuinely you know I don’t wanna harm myself. I don’t want to harm my children at all, but I just sometimes wish that I could go into a zone.

She stated that the interview itself was helpful for her to express how exhausted she was about bearing her day-to-day responsibilities. She described fatigue and feelings of being overwhelmed:

“You’re probably helping me right now, but it’s like damn, just in a little bit- I always have you tell people if I use a timer in my head that’s ticking every time I turn around, I gotta do this. I’m on this. I got- and I’m just- I’m tired of being tired.

Another participant reported that through her own experience, she recognized postpartum depression as a real phenomenon:

So it’s like, it’s a lot, and I just hope that anybody that does go through it just is, strong, clear-minded, level, because it’s a lot, but the baby is, the baby is what makes postpartum disappear. Okay, that’s the best part. That’s the prize at the end. And when I look at them, I can forget all about everything.

The notion that the baby makes one forget about physical or emotional trauma related to birth is a deeply embedded cultural expectation that may make both disclosing postpartum depression and seeking care difficult.

### 3.2 Theme 2: Self-Blame Around Postpartum Mental Health Sequelae

A second theme that emerged was participants’ self-blame around postpartum depression.

One of the interviewees expressed her frustration around postpartum depression symptoms of fatigue and feeling overwhelmed despite being a single mother of three children:

“That’s where my mental frustration comes from, because I’m tired of complaining of being tired, because generally I how I feel I should not be physically tired of… of doing- of doing a routine that I’m comfortable with doing, the routine that I set. You know my frustration comes from, you know- Just the type of person that I am, I- I’m talking and working. I’m trying to learn how to be selfish and just tackle and do what I gotta handle and do, because I feel like I get overwhelmed when I’m taking on so much-”

The self-doubt about the ability to parent, specifically as a single mother, was also evident in other participants as a reason for “personal failure” contributing to postpartum depression:

I wasn’t thinking about like killing myself or anything like that, but I would just feel down, think about like “Am I good enough to be like my son’s parent?” like his dad is not around so you know, I was just, I was just thinking about that too much in him having a good life with just having me so that would like put me down a little bit so that’s what caused my postpartum.

The same participant initially did not believe postpartum depression was **“real,”** and attributed the illness to personal or character deficiencies, until she started to experience it herself:

Postpartum, I didn’t believe it was real at first, I really just thought it was just some way that moms and women just made an excuse just to be, in my eyes, just be the bad person they were, or to let the feelings out that they had. It took me to, to be to go through it with my first child and start to, I feel it sometimes because I don’t have really bad postpartum, but I did go through postpartum, and I’m going through it again now.

Another participant reported not even being able to tell her partner due to the expectation that she should handle all this herself:

He honestly didn’t even know I had post-partum just because I was crying so much by myself in the room. When he did find out I had it, he was more so, “Why aren’t you coming to me and confiding in me?” I just had to pull myself out of it because I just wanted to deal with that myself.

Lack of comfort disclosing depression to social supports might have increased the intensity of emotion and prolonged and enhanced feelings of isolation for this patient.

### 3.3. Theme 2: Fear Admitting Postpartum Mental Health Sequelae to a Health Care Provider

A third theme was fear around reporting symptoms consistent with postpartum depression to a healthcare provider. Two participants specifically reported concerns around their healthcare team alerting child protective services of their depression and risking their child or children being removed from their care.

#### Participant 1:

Actually, because my sister, I have a lot of siblings. My sister, [name], ended up doing it. but they like- they ended up putting her in like, what is it called like? Not a psych ward, but like basically where you go and they like it’s like whenever you’re dealing with depression basically and that’s what they did to her I just don’t know the name of the hospital or whatever, and they did that to her. Whenever she was writing on her paper with her baby. But that was about 2 years ago. I don’t know if they’ve changed it. But I dealt with CYS my whole life and I’m just afraid of, you know that coming again I can’t deal with that. I- I just can’t deal with that. I’m scared. I’m just scared.

#### Participant 2

But when it would come to me like going to his visits, you know they have the papers that talks about postpartum depression. I’ll just put I’m okay for all of them because I was scared that they were gonna take my child or something.

The first participant continued that she had a sister who was hospitalized for reporting symptoms of postpartum depression and that her sister was placed in a facility where she was unable to keep her child.

One of the participants also alluded to her race as a reason why she was more likely to have child protective service involvement:

Cuz- I don’t know- I didn’t want them to think we- usually, when some moms get depressed they think they’re going to hurt your children and stuff like that, so it was just-I’mma be honest, because I’m black I thought that-, if I told them that I’m depressed that they will try to take my daughter from me and I didn’t- I just didn’t want that risk.

### 3.4. Theme 4: Social Support and Affirmation as Critical Elements in Coping with Postpartum Mental Health Sequelae

The fourth theme was that social support and affirmation appeared to have a positive effect on coping with postpartum mental health sequelae, while the absence of social support and affirmation had a negative effect.

A participant spoke candidly about her exhaustion parenting three children as a single mother despite her own mental fortitude:

I don’t like to think about it but I get reminded every day like damn, you know, [I] had the babies and I’m by myself. Just one thing you know, feeding them every day by myself is one thing- washing them up because that’s something that I was doing. I’m used to doing, I was gonna do that, but it just like- like damn every single day like I’m here by myself. I’m doing this by myself, you know, that sucks!

One participant, also a single mother, explained that she received positive affirmations from her mother and grandmother, which helped to improve her mood postpartum:.

My mom always told me, my grandma always tells me, anybody around me that I have around my son, they would tell me like you’re a good mom you’re doing real good for his age like, I was getting that a lot. Especially when my postpartum that’s another thing that like uplifted me like just be[ing] told I’m a good mom without having any help, except for my mother.

Another participant cited her partner as important support, assisting her to contend with her depressive symptoms, though she had endorsed earlier in the interview her reluctance in disclosing her symptoms to her partner:

I had a lot of postpartum depression, only because it was like I’m a young mom, I didn’t know what I was—I knew what I was doing because I had past experience in care of kids living with me. It’s just by myself, like I’m home with the baby—not by myself; I had him too, and he was a lot of help—

The themes that emerged from these interviews may have particular significance for diagnosing and treating postpartum mental health sequelae from a strengths-based approach–one that leverages the protective factors young mothers already have and one that also relies less on disclosure than on connection to therapeutic resources.

## 4. Discussion

This study examined pregnancy and birthing experiences of Black and Biracial adolescent and young adult birthers and determined several themes related to fears disclosing postpartum depressive symptoms. Examining the prevalence of depressive symptoms in perinatal populations is imperative to improve maternal and child health outcomes in the US. Children of untreated depressed mothers are more likely to have high risk for behavioral inhibition, poor cognitive functioning, emotional maladjustment, violent behavior, externalizing disorders, and psychiatric and medical morbidities in adolescence [18–26]. Additionally, mothers with untreated PPD have increased risk for substance use disorders [27], social relationship problems [28], breastfeeding problems [29], and persistent depression [30] compared with women who have received treatment.

The preponderance of unprompted discussions around postpartum mental health sequelae that emerged in nearly half our subjects suggests that Black and Biracial adolescent and young adult (AYA) mothers are experiencing depressive symptoms but may be under-reporting struggles with mental illness to clinicians and on screening forms. This finding is consistent with the results of a pilot intervention study of low-income Black mothers in New York City where patients were reluctant to acknowledge the impact of stress and mental health on screening tools. This is problematic because there is evidence that PPD is highest among 18–24 year old women, especially those who are first-time mothers. [31]. Further racial disparities exist both in the prevalence of PPD and the receipt of postpartum care, with Black and Hispanic women having higher rates of PPD [8] compared to white women, and less connectivity to care [9].

Mothers often attributed their depressive symptoms to personal weakness rather than illness. This theme of self-blame emerged in our participant narratives where postpartum depression “did not exist” or was attributable to personal failure. Our participants were also quick to shift blame away from their children as sources of pressure or reasons for their feelings. Social pressure for new mothers to bear the physical and mental sequelae of childbirth are obviously not new, but the nuances of how this may manifest in Black and biracial women may be instructive. Although Black women have higher prevalence of maternal mental health sequelae, including postpartum depression and anxiety, 1 maternal mental health issues among Black women are largely underreported, and symptoms often go unaddressed. In one study, Black mothers were asked “what do you do when you feel down in the dumps?” and the overwhelming majority, 63%, employed strategies that typically denied, masked, or suppressed their emotions rather than strategies which acknowledged symptoms, treating causes, or seeking professional help [32].

Additionally, the mythology around the “strong Black woman” (SBW) may be implicated in this expectation that Black women bear their mental illness alone. In one quantitative study by Watson and Hunter (2015) [33], the SBW schema positively predicted depressive symptoms. Another study [34] also found a positive association between the SBW schema and depressive symptoms and additionally elucidated that self-silencing as the link between the SBW schema and depression. The stereotype schema which may predispose young Black women to minimize postpartum mental health sequelae is exacerbated by the intersectionality of being a young Black mother in a culture which does not often favorably view Black mothers or young mothers. This makes capturing information around postpartum mental health sequelae for this population extremely difficult, though our experience interviewing our participants certainly revealed this to be a substantial and unmet need. Pittsburgh Healthy Start and the Infant Health Equity (IHE) Coalition, which includes local community perspectives and experiences, have suggested replacing or supplementing paper mental health screenings with one-on-one trusted maternal and child health workers, which aligns with the narratives of several of our participants [35].

It is imperative that mental health researchers and professionals develop targeted strategies that improve the comfort and remove barriers that dissuade young Black and biracial mothers from disclosing postpartum mental health sequelae and to normalize seeking appropriate treatment. Strategies that may be helpful considering the themes identified in this study would be greater inclusion of Black and biracial doulas, who are often seen as non-judgmental advocates, during pregnancy, as well as continued screening for postpartum mental health sequelae well beyond the six-week postpartum checkup.

Additionally, as many birthing people forgo their six-week checkup, pediatricians may be critical advocates and touchpoints for rescreening of postpartum depression in mothers further out from the infant’s birth. Additionally, it may be important to identify community support, as many participants in this study reported social isolation as a result of being a young mother. Centering groups, which offer birthing people a cohort of peers during their pregnancy as well as new parent support groups, can create community for young mothers navigating their new roles. Other interventions like early intervention and nurse visiting programs have also demonstrated efficacy in addressing disparities among adolescent mothers [36].

### Strengths and limitations

One of the benefits of our study was that the research team did not have a clinical role in the care of any participant included in the study. This offered distance from their clinical care that may have increased their comfortability disclosing mental health concerns. Many participants were no longer in their postpartum period at the time of the interview; this distance from the timing of their birth may have also increased comfortability in reflecting and disclosing postpartum mental health sequelae as something they had overcome. There were also some limitations, including the lack of inclusion of questions about perinatal mental health and postpartum depression specifically. Given the dearth of research on postpartum depression in Black and biracial AYA, more research is needed to better understand how to meaningfully care for this population.

### Conclusion

Black and biracial adolescent and young adult mothers may under-report depressive symptoms in the postpartum period due to structural and social barriers. Eliminating these barriers and improving the acceptability of reporting depressive symptoms for minority young women after experiencing pregnancy is vital to improve perinatal and infant health overall. The narratives and insight from participants in this study suggest the need to better understand social and historical reasons why Black and biracial AYA mothers may be unlikely to report postpartum depression to develop appropriate interventions to support a healthy pregnancy and postpartum period.

## Figures and Tables

**Table 1.** Participant characteristics

Age (years)	n
16–17	3
18–19	6
20–21	7
22–23	9
Race	
Black	22
Biracial	3
Pregnancy History	
First pregnancy	16
First birth	9
Still in high school	6
Employment	
Part or full-time employment	6
Not employed or enrolled in school	13
Home Life	
Live with parents	10
Live alone (with child/ren)	9
Living with father of the baby	4
Living with another relative	1
Other (unspecified)	1

## Data Availability

All data generated or analyzed during this study are included in this published article and its supplementary information files in the form of tables. Raw manuscripts of the qualitative interviews themselves are included in the supplementary le.
